# Infection with mosquito-borne alphavirus induces selective loss of dopaminergic neurons, neuroinflammation and widespread protein aggregation

**DOI:** 10.1038/s41531-019-0090-8

**Published:** 2019-09-13

**Authors:** Collin M. Bantle, Aaron T. Phillips, Richard J. Smeyne, Savannah M. Rocha, Ken E. Olson, Ronald B. Tjalkens

**Affiliations:** 1 Department of Environmental and Radiological Health Sciences, Colorado State University, Fort Collins, CO 80523 USA; 2 Arthropod-Borne and Infectious Disease Laboratory, Department of Microbiology, Immunology, and Pathology, Colorado State University, Fort Collins, CO 80523 USA; 3 Department of Neuroscience, Vickie & Jack Farber Institute for Neuroscience, Thomas Jefferson University, Philadelphia, PA 19107 USA

**Keywords:** Parkinson's disease, Neuroimmunology

## Abstract

Neuroinvasive infections with mosquito-borne alphaviruses such as Western equine encephalitis virus (WEEV) can cause post-encephalitic parkinsonism. To understand the mechanisms underlying these neurological effects, we examined the capacity of WEEV to induce progressive neurodegeneration in outbred CD-1 mice following non-lethal encephalitic infection. Animals were experientally infected with recombinant WEEV expressing firefly luciferase or dsRed (RFP) reporters and the extent of viral replication was controlled using passive immunotherapy. WEEV spread along the neuronal axis from the olfactory bulb to the entorhinal cortex, hippocampus and basal midbrain by 4 days post infection (DPI). Infection caused activation of microglia and astrocytes, selective loss of dopaminergic neurons in the substantia nigra pars compacta (SNpc) and neurobehavioral abnormalities. After 8 weeks, surviving mice displayed continued loss of dopamine neurons in the SNpc, lingering glial cell activation and gene expression profiles consistent with a neurodegenerative phenotype. Strikingly, prominent proteinase K-resistant protein aggregates were present in the the entorhinal cortex, hippocampus and basal midbrain that stained positively for phospho-serine129 α-synuclein (SNCA). These results indicate that WEEV may cause lasting neurological deficits through a severe neuroinflammatory response promoting both neuronal injury and protein aggregation in surviving individuals.

## Introduction

Parkinson’s disease (PD) is characterized by loss of voluntary motor control due to the degeneration of dopaminergic neurons of the substantia nigra pars compacta (SNpc), associated with oxidative stress, glial cell activation and α-synuclein protein aggregation. Therapies for PD are limited by an insufficient understanding of both the etiological factors and the pathological mechanisms influencing disease progression. The etiology of PD remains elusive but it is likely linked to interactions between genetic risk-factors, age and environmental stressors, including infectious agents.^1^ Viruses, in particular, have been linked to PD and there have been several reports of parkinsonism observed among human survivors of encephalitic viral infection.^2^ Exposure of the human central nervous system (CNS) to selected viruses have been shown to induce a phenotype that mimics the neuropathology and neurological dysfunction observed in cases of sporadic PD, and increasing evidence implicates viral infection of the CNS as a potentiating factor in multiple neurodegenerative diseases.^3–5^ For example, following the 1918 “Spanish Flu” pandemic, nearly every patient who had an acute episode of encephalitis lethargica went on to develop post-encephalitic parkinsonism, a condition with neurological symptoms that closely resemble PD.^2,6–8^ In addition, many neurotropic mosquito-borne viruses, including the West Nile virus (WNV), Japanese encephalitis virus (JEV), and St. Louis encephalitis virus have been shown to induce PD-like symptoms in humans and rodents.^9^ Recent evidence has suggested that over 50% of patients who survive neuroinvasion with WNV exhibit a neurodegenerative phenotype later in life.^10^

Older reports indicate that western equine encephalitis virus (WEEV), another mosquito-borne alphavirus, may be capable of inducing parkinsonism in humans following encephalitic infection and in a more recent report, 6 of 25 patients from a Colorado epidemic of WEEV presented with a “parkinsonian syndrome” with severe, progressive neurological sequelae.^11–13^ These symptoms included resting tremor, intellectual deterioration, and cogwheel rigidity,^13^ which persist long after resolution of acute infection with WEEV. This may be due to the chronic presence of viral RNA, because alphavirus RNA can persist in mouse brain 3–17 months after acute infection and could therefore induce a sustained neuroinflammatory reaction.^11^ Alphaviruses alter expression of transcripts associated with interferon signaling and unfolded protein responses, and we have previously shown that infection with WEEV induces expression of inflammatory cytokines and chemokines in mouse brain and serum, including MCP-1, IL-12, IFN-γ, and TNF-α, that are also associated with PD.^14–16^ Furthermore, alphaviruses inhibit mitochondrial bioenergetics, thereby depressing cellular ATP content and inducing oxidative stress, which are associated with neuronal injury in PD and related neurodegenerative disorders.^17^ Although these studies suggest that a single encephalitic infection with WEEV can induce post-encephalitic parkinsonism in the surviving host, the pathogenic mechanisms remain unclear.

To identify mechanisms by which WEEV and related neurotropic alphaviruses cause such persistent neurological deficits following encephalitic infection, we examined the capacity of WEEV to induce progressive neurodegeneration after a single intranasal inoculation in CD-1 mice. We postulated that encephalitic infection WEEV would trigger neuroinflammatory activation of microglia and astrocytes that contributed to neuronal injury and neurobehavioral abnormalities. We demonstrate that intranasal inoculation with recombinant WEEV expressing firefly luciferase (“McFly”) induced a consistent and broad encephalitic infection in outbred CD-1 mice and caused robust glial activation and selective loss of dopaminergic neurons in the SNpc. Morbidity and mortality by intranasal infection ocurred within 4 days but could be attenuated by passive immunotherapy using polyclonal antibodies to the ectodomain of the WEEV E1-glycoprotein to control the extent of infection.^18,19^ Surviving mice completely cleared the infection by 8 weeks and were examined for pathological features associated with neurodegeneration in multiple brain regions, including cortex, hippocampus, and the basal midbrain. Following inoculation with McFly and immunotherapy, surviving mice displayed selective loss of dopaminergic neurons in the substantia nigra, as well as behavioral abnormalities and persistent activation of glial cells. In addition, we detected the formation of proteinase K-resistant α-synuclein protein aggregates and a gene expression profile consistent with a progressive neurodegenerative phenotype. In summary, these data indicate that CNS infection with WEEV in wild-type mice causes a pattern of neuronal injury, glial activation and protein aggregation that resembles pathological features of PD and related disorders, which could explain the diagnosis of post-encephalitic parkinsonism in individuals actually infected with WEEV and similar alphaviruses.

## Results

### Infection with recombinant WEEV causes significant neuroinvasion and rapid dissemination from the olfactory bulb throughout the brain

**Fig. 1:**
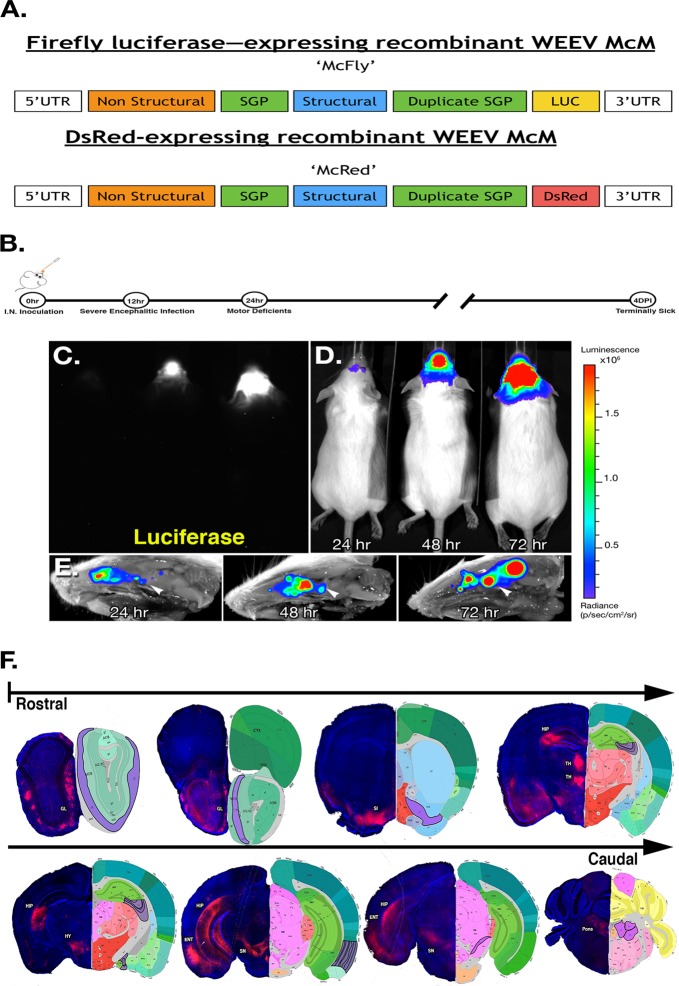
Regional specificity of recombinant Western equine encephalitis virus following intranasal infection. **a** Schematic diagram illustrating the structure of vectors for each recombinant virus used in these studies. Subgenomic promoter (SPG), untranslated region (UTR). **b** Schematic diagram illustrating treatment regimen with WEEV. **c** Raw image of luciferase activity in McFly-infected mice (left-24 HPI, middle-48 HPI, right-72 HPI). **d** Pseudo-colored image of luciferase activity overlaid onto whole body images during the timecourse of infection. **e** Ex vivo sagittal hemispheric images of luciferase activity at specific times post infection demonstrating progressive spread of WEEV from the olfactory bulb to caudal brain regions. **f** Six-week-old CD-1 mice were administered 1 × 10^4^ PFU of DsRed producing WEEV (McRed) via intranasal inoculation and euthanized 4 days post infection. Brains were cryosectioned coronally and dsRed + infected nuclei were labeled (purple) with reference to the allen brain atlas. Individual nuclei with presence of Dsred were labeled: GL (glomerular layer), SI (substantia innominate), MB (mammillary hippocampus (HIP), thalamus (TH), entorhinal regions of the cortex (ENT), and pons

Recombinant WEEV vectors, McFly and McRed (Fig. 1a), were constructed to express reporter proteins as markers of infection (firefly luciferase and DsRed), using a duplication of the subgenomic promoter sequence. We have previously imaged the entry and spread of alphavirus into the CNS resulting from intranasal and peripheral infection.^9,20,21^ Although both routes of inoculation lead to midbrain infection, we used the intranasal route of infection in the current study due to its efficiency in establishing CNS infection. In a preliminary comparison of different WEEV viral strains, medium-virulence (Montana-64) and high-virulence (McMillan) strains of WEEV, we found that the high-virulence strain resulted in significant loss of dopamine neurons in the SNpc within 4 days of infection (Supplementary Fig. 1). This strain was therefore used as the basis for constructing McFly and McRed vectors.

To identify the extent and spread of WEEV throughout the neuraxis, 6-week-old CD-1 mice were intranasally inoculated with McFly at a concentration of 1 × 10^4^ PFU. Luciferase activity was measured in live, intact animals each day for the duration of the infection (Fig. 1c, d). Mice were scored by a clinical scoring system in 12-h increments to assess the severity of the infection on neurobehavioral function. Healthy mice received a score of zero, mice with ruffled received a score of one, mice with slight lethargy, hunched posture and obvious irritable received a score of two, very irritable mice with obvious motor deficits received a score of three, and moribund mice received a score of four. All mice challenged with McFly showed robust luciferase activity and apparent neurobehavioral and motor deficits by 24 h post infection, ranging from slight lethargy and hunched posture to severe hypokinesia and ataxia. Without immunotherapy, mice were unable to survive more than 96 h because of the severity of the encephalitic infection (Fig. 1b, c, d).

To further characterize the distribution of McFly following intranasal infection, a second cohort of mice was infected and sectioned along the sagittal axis for ex vivo imaging. McFly appeared to propagate from the olfactory bulb to the cortices and dorsal basal ganglia, as well as the basal midbrain encompassing the substantia nigra (SN), by 72 h post infection (Fig. 1e).

To better track WEEV at the microscopic level and identify the individual nuclei infected following intranasal infection, we employed the DsRed-expressing McRed virus. Analysis of brain tissue from mice infected with McRed revealed consistent viral dissemination in all animals. Brains processed for CLARITY were sectioned from the rostral olfactory bulb to the caudal cerebellum. At 96 h, McRed-infected multiple nuclei involved in neurodegeneration, the glomerular layer of the olfactory bulb (GL), substantia innominate (SI), hippocampus (HIP), thalamus (TH), entorhinal regions of the cortex (ENT), pons, and most notably, the substantia nigra pars compacta (SNpc) (Fig. 1f). Whole-brain 3D reconstructions of McRed-infected brains were also performed following subcutaneous and intranasal infection to identify the global distribution of virus throughout the CNS (Supplementary Fig. 2). Notably, intranasal infection with WEEV/McRed resulted in a pattern of viral spread that largely bypassed the striatum but still infected neuronal soma in the substantia nigra and ventral tegmental area.

### Intranasal inoculation with WEEV causes selective dopaminergic neuronal loss and glial activation within 4 days of infection

**Fig. 2:**
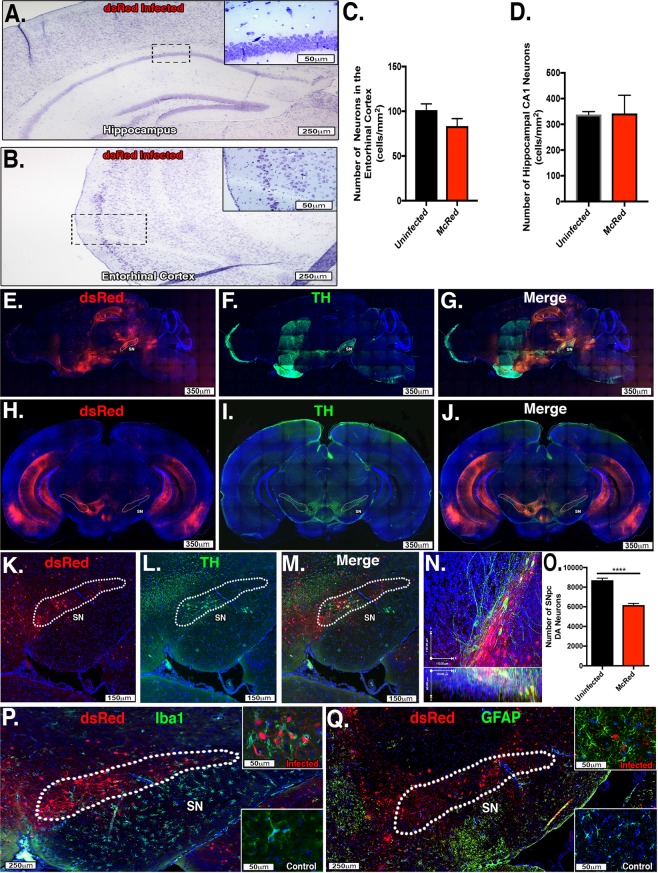
dsRed-expressing WEEV (McRed) shows strong tropism for dopaminergic neurons and causes selective dopaminergic neuronal loss and glial activation following intranasal inoculation. **a**–**d** Neuronal densities in hippocampus and entorhinal cortex were examined at 4 DPI following intranasal infection with McRed. No significant neuronal loss was detected in the hippocampus (CA1) or the entorhinal cortex (338 ± 6.557 vs. 342.8 ± 9.259, *p* = 0.7142 and 101.3 ± 6.96 vs. 90.4 ± 18.1, *p* = .6730) (*N* = 4/group). **e**–**g** Sagittal and coronal sections from CLARITY processed brains (**h**–**j**) were immunostained for TH + dopaminergic neurons. High-magnification confocal images (**k**–**n**) demonstrate co-localization of DsRed (red) and TH + DA neurons (green). White dotted regions delineate the substania nigra pars compacta (SN). **n** *X* and *Y* coordinates in top panel with *X* and *Z* coordinates in lower panel. **o** Number of DA neurons in SNpc were examined with 3D design-based stereology 4 DPI. A 29% loss of DA was observed between treatment groups. (29% loss, 8707 ± 202.2 vs. 6180 ± 162.6). Intense staining for IBA1-positive microglia (green) (**p**) and GFAP-positive astrocytes (**q**) was noted at 4 DPI in the SN following infection with McRed when compared with aged matched controls (bottom right of panels). White dotted region delineates the substania nigra pars compacta (SN) (*N* = 3/group). ****p < 0.0001

After characterizing the neuroanatomical propagation of WEEV following intranasal infection (Fig. 1f), we next wanted to determine if WEEV infection induced neuronal loss in infected brain regions. Intranasal infection with McRed induced significant infection in the hippocampus (CA1) and entorhinal cortex by 4 DPI (Fig. 1f) but neuronal counts of these brain regions showed no significant neuronal loss when compared with age-matched controls (Fig. 2a–c; hippocampus, 338 ± 6.557 vs. 342.8 ± 9.259, *p* = 0.7142 and entorhinal cortex, 101.3 ± 6.96 vs. 90.4 ± 18.1, *p* = 0.6730). To further characterize the neuronal tropism of WEEV, coronal and sagittal sections were stained with anti-tyrosine hydroxylase (TH) and imaged by fluorescence microscopy for co-localization with McRed, which strongly colocalized with TH + dopaminergic neurons in the SNpc (Fig. 2e–n). 3D confocal microscocy of the SNpc revealed widespread expression of dsRed in neurons throughout this nucleus (Fig. 2n). Stereological counts of neurons within the SNpc showed significant loss of TH + dopaminergic neurons at 4 DPI (29% loss, 8707 ± 202.2 vs. 6180 ± 162.6, *p* < 0.0001) when compared with age-matched uninfected controls (Fig. 2o). In addition, marked activation of microglia and astrocytes was evident in McRed-infected brains, with microglia phagocytosis of dsRed-positive cells by 4DPI (Fig. 2p). Hypertrophy and proliferation of GFAP + astrocytes was noted proximal to dsRed-positive neurons in the SNpc (Fig. 2q).

### Anti-viral immunotherapy attenuates CNS infection with WEEV and facilitates viral clearance by 8 weeks post infection

**Fig. 3:**
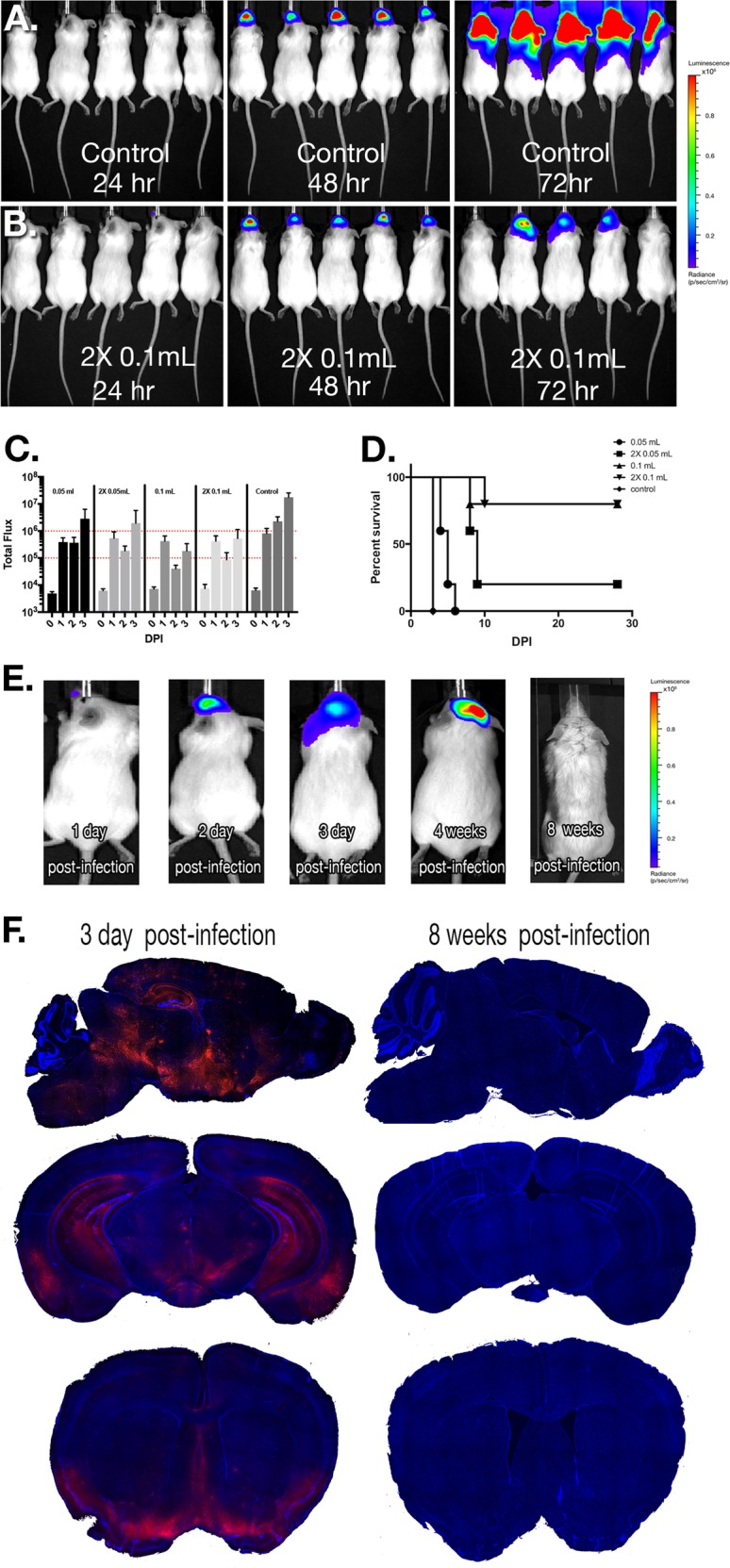
Anti-E1 immunotherapy in WEEV-infected CD-1 mice rescues from lethal infection and facilitates clearance of virus by 8 weeks post infection. **a**, **b** Mice were infected with McFly and received either a mock treatment (naive pre-immune serum) or anti-E1 immunotherapy (anti-E1 polyclonal rabbit immune serum). Bioluminescence measurements were acquired every 24 h following inoculation. **c** Immunotherapy treatment was optimized to achieve consistent CNS infection without mortality, demarcated by red lines indicating the upper and lower range of acceptable luciferase activity. For each treatment regimen, the measured luciferase activity is shown for the head region. Volumes indicate amount of immunized rabbit serum administered. 2X means the animals in that group received doses of immune serum at both 12 and 48 h post infection. **d** Survival curves for the different treatment regimens employed. **e**, **f** Following treatment with immune serum at 12 and 48 h post infection, regimen after intranasal infection with McFly and McRed, animals completely cleared virus by 8 weeks post infection

Given that intranasal inoculation with WEEV caused a severe encephalitic infection with rapid propagation of virus throughout the CNS, we employed passive immunotherapy to prevent mortality in order to characterize the long-term neurological consequences of infection with this alphavirus. The immunotherapy regimen targeting the E1-glycoprotein of WEEV facilitated viral clearance in infected mice, as we reported in previous studies.^21,22^ We optimized the immunotherapy treatment regimen with McFly virus to generate an inoculation protocol that led to active spread of the virus into the midbrain without necessitating euthanasia (Fig. 3). Infection of CD-1 mice with McFly was followed by either one treatment at 12 h or two treatments at 12 and 48 h with either 0.05 or 0.1 mL of immune serum, or with naive control serum (Fig. 3a, b). Photon counts of luciferase activity over the first 3 days of infection demonstrated that 2 × 0.1 mL of immune serum resulted in the best overall profile of luciferase expression without incapacitating the animal (Fig. 3c). Animals with luciferase activity measurements in excess of 10^6^ photons/sec became severely ill, were euthanized and not used for analysis. Animals with luciferase activity measurements <10^5^ photons/s were considered not to have established a CNS infection and were similarly not used for analysis. Any animals having luciferase activity within the defined acceptable range of photon counts (demarcated by the dotted red lines in Fig. 3c) were kept in the study. Survival curve analysis of the various treatment strategies showed that two doses of 0.1 mL treatment yielded the highest survival percentage (Fig. 3d). Optimization of the treatment regimen indicated the presence of luciferase activity in the brain for up to 28 days following inoculation with McFly virus (Fig. 3e), with no luciferase activity detected at 8 week post infecxtion. In parallel studies with McRed virus, mice were similarly treated with passive immunotherapy and brains imaged following CLARITY to detect the presence of dsRed. By 8 weeks post infection there was no detectible dsRed signal all virus appeared to be cleared from the brains of infected mice (Fig. 3f, h). This finding is similar to previous results from our group using in vitro neutralization and plaque assays with infected brain homogenates, in which WEEV was neutralized with anti-E1 immunotherapy and completely cleared from infected mouse brain following IP treatment with anti-E1 immunoserum.^21,22^

### Mice rescued from WEEV infection using immunotherapy display loss of dopaminergic neurons with associated motor deficits

**Fig. 4:**
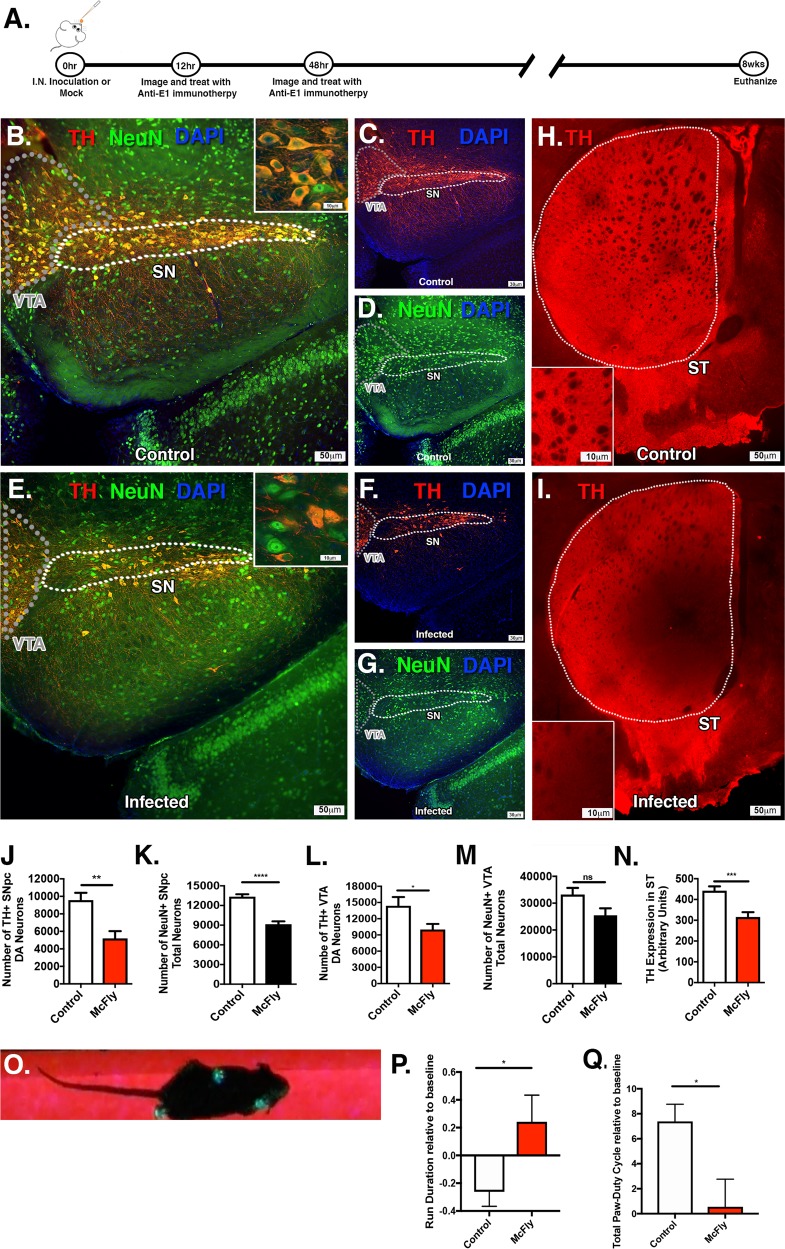
Following intranasal infection with WEEV and immunotherapy, surviving mice develop progressive dopaminergic neuronal loss and motor deficits. **a** Six-week-old CD-1 mice were intranasally (I.N.) inoculated with luciferase-expressing WEEV (McFly) or saline vehicle control and treated with anti-E1 polyclonal antiserum at 12 and 48 h post infection and euthanized 8 weeks later. Outlined regions indicating the VTA and SN were used for quantification. **b**–**g** Immunofluorescence of DA neurons (red) and total neurons (green) of control or infected. **h**, **i** DA terminals from the striatum of control and infected mice with representative high-magnification images. White dotted line indicates the region used for quantification. **j**–**n** Quantitative assessment of DA neurons and total neurons in the SNpc and revealed a 38% loss of TH + dopaminergic neurons (9774 ± 494.5 vs. 6009 ± 374.5) and a 31% loss of NeuN + total neurons (13347 ± 365.6 vs. 9141 ± 436.1) in the SNpc. Dopaminergic neurons in the VTA were decreased by 30% following infection (14,388 ± 1624 vs. 10,002 ± 1026), without significant loss of NeuN + total neurons (33,216 ± 2463 vs. 25,488 ± 2595). **n** DA terminal mean intensity reduction of 29% when compared with control. **o**–**q** Infected mice exhibited an increased run duration when traveling a fixed distance, along with a decreased duty cycle relative to control mice (0.242 ± 0.1912 vs. −0.2618 ± 0.1044 and 0.5509 ± 2.211 vs. 7.383 ± 1.378) when measured with a quantitative gait analysis system. All measurements were compared with baseline control values for each individual mouse (*N* = 6/group). *p < 0.05, **p < 0.005, ***p < 0.0005, ****p < 0.00005

After optimizing our infection and treatment regimen with anti-E1 immunotherapy, we next examined the neuropathology, dopaminergic neuron loss and motor deficits following encephalitic infection with McFly at 8 weeks post infection, after complete clearance of virus from the brain (Fig. 4). Mice were infected intranasally with McFly or mock-infected with saline and treated with immunotherapy at 12 and 48 h. Surviving mice were euthanized at 8 weeks post infection (Fig. 4a). To determine whether infection with McFly induced selective loss of dopamingernic neurons in the SNpc or generalized loss of dopaminergic neurons in the basal midbrain, dopaminergic neuronal loss was assessed in the (SNpc) and ventral trigeminal area (VTA), as well as dopaminergic terminals in the striatum (ST). Analysis of infected mice revealed a significant loss of dopaminergic soma in both medial and lateral aspects of the SNpc, with apparent necrotizing morphology of remaining dopaminergic neurons, coupled with deteriorating projecting fibers to the substantia nigra pars reticulata (SNr) (Fig. 4b–g). Loss of dopaminergic neurons in the VTA was also noted. Quantitative analysis of infected brains revealed a 38% loss of TH + dopaminergic neurons (9774 ± 494.5 vs. 6009 ± 374.5, *p* < 0.005) and a 31% loss of NeuN + total neurons (13347 ± 365.6 vs. 9141 ± 436.1, *p* < 0.00005) in the SNpc. Dopaminergic neurons in the VTA were decreased by 30% following infection (14388 ± 1624 vs. 10002 ± 1026, *p* < 0.05), without significant loss of NeuN + total neurons (33216 ± 2463 vs. 25488 ± 2595, *p* < 0.0005) (Fig. 4i, l). Dopamine terminal loss was also observed in the ventromedial striatum when compared with age-matched controls receiving only immunotherapy (Fig. 4m), with a 29% decrease in fluorescence staining intensity relative to control animals (442.1 ± 21.06 vs. 315.8 ± 22.99 arbitrary fluorescence units).

Surviving mice showed apparent neurobehavioral deficits consistent with deprecations of dopaminergic function that included postural instability, tremors, dyskinesia, and rigidity (S3). In addition, quantitative gait analysis was conducted using a real-time video gait analysis system (Fig. 4n). All measurements where compared back to baseline control measures for each individual mouse. Infected mice exhibited obvious lethargy and an increased run duration when traveling a fixed distance, along with a decreased duty cycle relative to control mice (0.242 ± 0.1912 vs. −0.2618 ± 0.1044 and 0.5509 ± 2.211 vs. 7.383 ± 1.378, *p < 0.005). Duty cycle is defined as the percentage of stance over the sum of stance and swing duration, and stance is defined as the time each paw is in contact with the glass trackway.^23^ Increased run duration and decreased duty cycle both indicate hypokinesia in surviving mice 8 weeks post infection with WEEV.

### Encephalitic infection with WEEV causes persistent activation of microglia and astrocytes in surviving host animals

A well-described clinical feature of post-encephalitic parkinsonism is microgliosis and astrogliosis throughout the CNS, mediated by damage associated molecular patterns (DAMP) and pathogen associated molecular patterns (PAMP) that are primarily expressed on glial cells.^24–26^

**Fig. 5:**
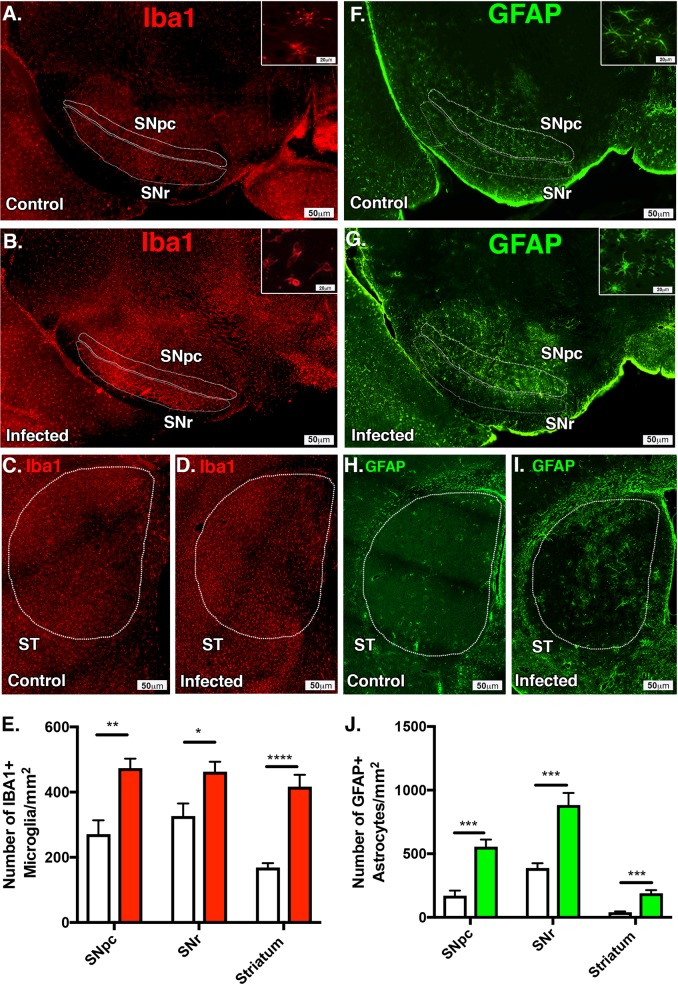
Persistent microgliosis and astrogliosis is present following encephalitic infection with WEEV. Six-week-old CD-1 mice were intranasally (I.N.) inoculated with luciferase-producing McFly virus or saline and intraperitoneally treated with anti-E1 polyclonal antibody at 12 and 48 h post infection and euthanized 8 weeks later. **a**, **b** White dotted region and gray dotted regions delineate the substania nigra pars compacta (SNpc) and substania nigra reticulate (SNr) used for microglia (IBA1, red) quantification. **c**, **d** Iba1-positive microglia were detected by immunofluorescence microscopy in control (A) and infected (B) CD-1 mice 1 week post infection. White dotted region indicates the striatum of control and infected animals. **e** Quantitative analysis of each respective brain region in control (white) and infected brains (red) revealed a 46% increase of IBA1 + microglia in the SNpc (499.5 ± 52.37 vs. 270.8 ± 42.27), a 31% increase IBA1 + microglia in the SNr (486 ± 42.55 vs. 337.3 ± 45.19), and a 40% increase of microglia in the ST (416.6 ± 36.37 vs. 248.6.3 ± 38.87). **f**, **g** White dotted and gray dotted regions delineate the substania nigra pars compacta (SNpc) and substania nigra reticulata (SNr) used for astrocyte (GFAP, green) quantification. **c**, **d** White dotted region indicates the striatum of control and infected animals. **j** Quantitative analysis of infected brains revealed a 69% increase of GFAP + astrocytes in the SNpc (555.1 ± 57.1 vs. 170 ± 40.84), a 56% increase in GFAP + astrocytes in the SNr (882.6 ± 94.91.55 vs. 494.8 ± 102.1), and a 79% increase of astrocytes in the ST (189.8 ± 24.29 vs. 40.07 ± 6.859) (*N* = 6/group). *p < 0.05, **p < 0.005, ***p < 0.0005, ****p < 0.00005

To characterize the extent of glial cell activation following infection with WEEV, we determined the relative number of microglia and astrocytes in the SNpc, ST, and VTA in mice at 8 weeks post infection (Fig. 5). We observed gliosis throughout the CNS in mice surviving encephalitic infection with WEEV but considering the selective loss dopaminergic neurons (Fig. 2), we decided to quantify gliosis throughout the nigrostriatal pathway solely. There was significant microgliosis in the SNpc, SNr, and ST with associated amoeboid/reactive microglial morphology 8 weeks post infection in surviving mice when compared with age-matched controls (Fig. 5a–d). Quantitative analysis of infected brains revealed a 46% increase of IBA1 + microglia in the SNpc (499.5 ± 52.37 vs. 270.8 ± 42.27, *p* < 0.005), a 31% increase IBA1 + microglia in the SNr (486 ± 4 2.55 vs. 337.3 ± 45.19, *p* < 0.05) and a 40% increase of microglia in the ST (416.6 ± 36.37 vs. 248.6.3 ± 38.87, *p* < 0.00005) (Fig. 5e). Astrogliosis was quantified using glial fibrillary acidic protein (GFAP). Infected animals exhibited significantly more GFAP + astrocytes in the SNpc, SNr, and ST with apparent hypertrophic/reactive morphology at 8 weeks post infection (Fig. 5f–i). Quantitative analysis of infected brains revealed a 69% increase of GFAP + astrocytes in the SNpc (555.1 ± 57.1 vs. 170 ± 40.84, *p* < 0.0005), a 56% increase in GFAP + astrocytes in the SNr (882.6 ± 94.91.55 vs. 494.8 ± 102.1, *p* < 0.0005), and a 79% increase of astrocytes in the ST (189.8 ± 24.29 vs. 40.07 ± 6.859, *p* < 0.0005) (Fig. 5j).

### Intranasal infection with McFly causes the formation of proteinase K-resistant α-synuclein plaques and a gene expression profile consistent with neurodegeneration

**Fig. 6:**
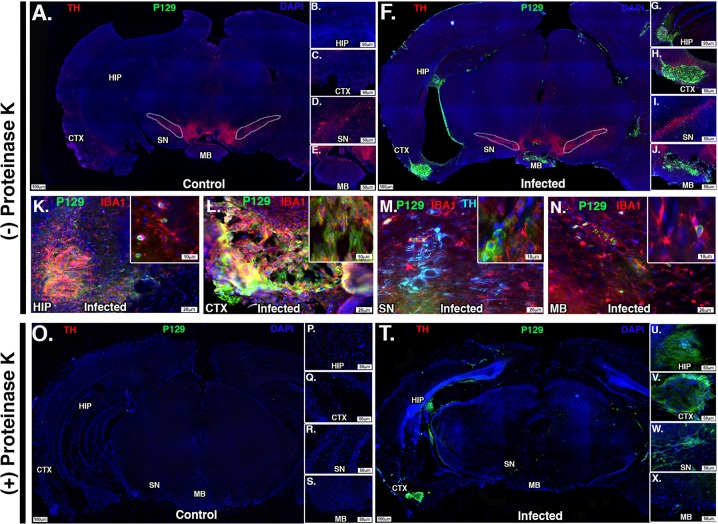
Surviving mice show formation of proteinase K-resistant α-synuclein aggregates in multiple brain regions. CD-1 mice were infected with McFly virus and treated with immunotherapy regimen and euthanized 8 weeks post inoculation. **a**–**j** Infected and control sections were stained with TH (tyrosine hydroxylase, red), P129 (phosphorylated α-synuclein 129, green), and DAPI 8 weeks post inoculation with McFly. **b**–**e** High-magnification images of uninfected control mice and **g**–**j** infected brain mice. White dotted region delineates the substania nigra pars compacta (SN). Individual nuclei positive for P129 staining were labeled as follows: HIP (hippocampus), CTX (cortex), and MB (mammillary body). **k**–**n** High-magnification images, ×20 magnification, showing P129 + plaques and co-localization with IBA1 + microglia in select nuclei. **o**, **x** Proteinase K-treated brain sections with complete degradation of TH depicting proteinase K-resistant α-synuclein aggregates (green). Also depicted are high-magnification images showing the presence of P129 + proteinase K-resistant plaques in infected mice (**u**–**x**) and the absence of P129 + plaques in uninfected control mice (**p**–**s**)

Previous studies have shown that encephalitic infection can induce the formation of α-synuclein aggregation following infection in humans and mice.^27–30^ To determine how infection with WEEV could modulate the aggregation of α-synuclein in outbred CD-1 mice, we examined brain sections from infected and control mice for expression of α-synuclein phosphorylated at Serine 129 (P129), a marker of the aggregated form of the protein.^31^ Selected sections were also treated with proteinase K to assess the formation of insoluble α-synuclein plaques. We observed prominent P129 + α-synuclein aggregate formation in the hippocampus, cortex, substania nigra and mammillary body in surviving mice (Fig. 6a–j). Acutely infected mice that were not treated with immunotherapy did not show P129 + α-synuclein aggregates at 4 DPI in any brain regions examined. Cells positive for P129 immunoreactivity appeared to co-localize primarily with IBA1 + microglia in all P129 + positive nuclei and did not appear to co-localize with TH + or NeuN + neurons in the substania nigra (Fig. 6k–n).

We then performed immunostaining in proteinase K-treated sections for TH and P129 to assess the formation of proteinase K-resistant aggregates in mice following intranasal infection with WEEV at 8 weeks post infection. Proteinase K treatment caused complete degradation of TH in both control and infected sections with no loss of DAPI nuclear staining. Immunostaining for P129 in treated sections revealed the presence of proteinase K-resistant α-synuclein aggregates in the hippocampus, cortex, substania nigra and mammillary body only in infected sections (Fig. 6o–x).

Considering the extensive distribution of α-synuclein aggregates throughout the CNS following infection with WEEV and since neurodegenerative mechanisms are likely to be common despite the sensitivity of the substantia nigra, we determined the transcriptional profiles using quantitative PCR arrays (qPCR arrays) on whole-brain homogenates to examine the expression of genes associated with a neurodegenerative phenotype (Supplementary Fig. 3). Transcriptional analysis was performed on mice having received one of the following treatments: animals receiving McFly virus and anti-E1 immunotherapy collected at 3 days post inoculation, control animals receiving mock infection (media only) collected at 3 days, animals receiving McFly virus and anti-E1 immunotherapy collected at 4 weeks post inoculation and control animals receiving mock infection and anti-E1 immunotherapy (as immunotherapy control) collected at 4 weeks post mock infection (Supplementary Fig. 3A). All results were normalized to mock-infected/IgG treated mice. The following RT² Profiler PCR Arrays were used for cDNA quantification: Mouse mTOR Signaling (Supplementary Fig. 3B), Mouse Parkinson’s Disease Array (Supplementary Fig. 3C), and Mouse Cell Death PathwayFinder (Supplementary Fig. 3D). Genes with statistically significant differences in fold-regulation, when compared with the mock-infected control animals, were graphed in ascending order. The gene expression profile at 3 DPI was consistent with previously characterized profiles following acute encephalitic infection with a mosquito-borne virus and appeared to be involved with innate anti-viral immune activation.^32^ When comparing the gene profiles at 3 DPI and 28 DPI, a shift in the pattern of gene expression was observed. At 28 DPI, genes more closely related to neurodegeneration were upregulated on the Cell Death array, including *Htt, Bcl2, Irgm1*, and *Akt1*, whereas and *Park7* and *Aldh1a1* were upregulated on the mouse PD array (Supplementary Fig. 4C, D). *Phospholipase D1* (*Pld1*)*, Phospholipase D2* (*Pld2*) and *Pten* were 4–6-fold upregulated in surviving host at 28 DPI, and have been implicated in neurodegenerative diseases and downstream signaling of ß-amyloid and *amyloid precursor protein* (*APP*) (Supplementary Fig. 4A).^33,34^ The PD array revealed sixfold upregulation of *Immune-related GTPase M* (*Irgm1*), which is associated with neuronal autophagy (Supplementary Fig. 4C).^35^ *DJ-1 protein* (*Park7*) was upregulated fourfold in surviving host mice as well. In addition, multiple genes involved in neurodegeneration, including *Huntington protein* (*Htt*) and *amyloid precursor protein* (*APP*), were also found to be upregulated fourfold in surviving host mice when compared with controls and acutely infected mice at 3 DPI. Additional genes that were uniquely upregulated in the 28 DPI group on the Cell Death and PD gene arrays appeared to be involved with calcium homeostasis and mitochondrial dysfunction.

## Discussion

Increasing epidemiologic and experimental evidence suggests that viral infection is a possible risk-factor for neurodegenerative diseases such as Parkinson’s disease (PD) and Alzheimer’s disease (AD).^5,27,36^ In the present study, we found that encephalitic infection with WEEV in outbred CD-1 mice by intranasal inoculation resulted in viral spread from the olfactory tracts to the basal midbrain. We did not detect infection within peripheral neurons of mice, based on in vivo imaging of mice infected with McFly, which suggests that WEEV infection in outbred CD-1 mice is highly specific to the brain. This finding is similar to previous studies that have shown Sindbis virus, another neurotropic mosquito-borne virus, can infect the spinal cord of mice but is unable to infect the peripheral nerves.^37^ This specificity to the CNS is likely due to the fact that peripheral neurons lack the amount of ER, polyribosomes, mitochondria, and oxidative environment observed in CNS neurons to facilitate alphavirus replication.^38–42^ In addition, the Braak staging system of human Parkinson’s disease demonstrates that α-synuclein pathology spreads in a stereotyped manner beginning in the enteric nervous system and olfactory bulb; and may be the result of an unknown pathogen.^18,19^ Notably, 7–8.3% of PD brains show Lewy body pathology in the SN without any dorsal motor nucleus of vagus (DMV) pathology indicating that olfactory spread may be independent of DMV involvement.^43,44^ Although the dorsal motor nucleus and gastrointestinal tract were completely void of pathology, we show that following intranasal inoculation, McFly rapidly disseminates from the olfactory bulb to the basal ganglia, similar to the progressive spread of α-synuclein pathology cases in sporadic PD (Fig. 1).

WEEV is a non-promoter driven virus and simple intranasal inoculation facilitates consistent viral propagation from the olfactory bulb to the entorhinal cortex, hippocampus and basal ganglia by 4 DPI (Fig. 1f). The mechanisms underlying the tropism of WEEV for these brain regions remains to be elucidated but the initial route of exposure and the neuroanatomical architecture certainly influence this regional specificity.^9^ McRed likely propagates transneuronally from the olfactory bulb through the entorhinal region and hippocampus and then replicates to the SNpc, in this respective order (Fig. 1f). We previously reported the entry and spread of alphavirus into the CNS resulting from intranasal and peripheral infection and demonstrated that both routes of inoculation lead to midbrain infection with differing distribution patterns (S2).^9,20,21,45^ Neuronal surface adhesion molecules like glycosaminoglycan heparan sulfate, as well as the unique oxidative intracellular environment of select neuronal populations have been cited as possible tropic factors.^46,47^ Previous studies have shown that the replicative protein of WEEV, nsP1 (non-structural protein 1), is sensitive to oxidative changes and needs a highly oxidative intracellular environment for viral replication, suggesting that RNA viruses may utilize oxidative stress induced during infection to control genome RNA capping and viral replication.^48^ The high oxidative environment of select brain regions such as the thalamus, entorhinal cortex, hippocampus and substania nigra likely contributes to the consistent dissemination of WEEV to these regions (Fig. 1).^49–52^

Similar to encephalitic infection with other neurotropic viruses such as coxsackie B4, Epstein-Barr virus, Japanese B encephalitis, and HIV, WEEV induced broad viral dissemination and gliosis but caused selective degeneration of neurons largely within the SNpc following acute infection at 4 DPI (Fig. 2).^27,45,53–61^ Although we did not observe α-synuclein aggregation at 4 DPI, the association seen between α-synuclein aggregation in the entorhinal cortex and hippocampus in surviving mice at 8 weeks post infection supports the assertion made in other studies that α-synuclein aggregation represents a direct or indirect neuroprotective and antimicrobial response.^5,27,62^ Whether α-synuclein directly induces neuroprotection by restricting viral replication or indirectly regulates gene expression of anti-viral cytokines, chemokines, and neuroprotective signaling molecules is unclear. It is also unknown whether viral-mediated loss of dopaminergic neurons following acute infection with WEEV is due in part to an increased neuroinflammatory response of microglia and astrocytes remains to be determined but we noted significant microglial phagocytosis of DsRed + SNpc neurons at 4 DPI (Fig. 2p). The removal of these damaged neurons by activated microglia is both a homeostatic mechanism but also likely related to broader inflammatory activation of glia that is neurotoxic, as is associated with the pathogenesis of PD and related disorders.^63–67^

To further characterize the long-term neurological consequences of encephalitic infection with WEEV, we optimized an anti-E1 immunotherapy regimen that facilitated viral clearance and host survival. Although viral RNA can act as a continued source of inflammation, imaging of luciferase and dsRed at 8 weeks post infection indicated that WEEV was completely cleared at this time point (Fig. 3f). Surviving animals showed motor deficits, loss of TH + dopaminergic neurons (38% loss) and NeuN + total neurons (31% loss), as well as significant loss of dorsomedial dopamine terminals in the striatum (29% loss) when compared with acutely infected mice without immunotherapy (Fig. 4). Interestingly, we observed equal levels of viral replication in the SNpc and VTA, although the dopaminergic neurons in the VTA appeared to be less affected, demonstrating the selective vulnerability of SNpc dopaminergic neurons.^68^

Considering that WEEV is completely cleared from the brain at 8 weeks post infection, the pathological features noted at this time point are likely due to chronic inflammatory activation of glial cells following infection (Fig. 5). Glial activation is a common feature of viral encephalitis, with astrogliosis observed in the frontal and temporal white matter in cases of von Economo’s disease and in post-encephalitic PD.^17,45,46^ Neuroinflammation is associated with all neurodegenerative diseases, characterized by inflammatory activation of both astrocytes and microglia.^69^ Here we saw persistent glial activation in the SNpc, SNr, and ST following encephalitic infection with McFly that likely contributes to the increased neuronal loss in surviving host animals. Neuronal injury from activation of microglia and astrocytes is consistent with that described in other encephalitis models, such as experimental autoimmune encephalomyelitis (EAE), that causes severe glial reactions and release of glial-derived neurotoxic factors.^70^ Similar to such models of encephalitis, we propose that innate immune inflammatory responses in glial cells are primarily responsible for the reactive gliosis observed after infection with WEEV.^71^ Following viral infection, astrocytes and microglia can adopt a long-lasting neurotoxic and proinflammatory phenotype that may help to combat infection but may also worsen neuronal degeneration.^69^ Consistent with this idea, it was previously reported that infection with WEEV in CD-1 mice resulted in increased cytokine and chemokine expression (CCL2, CXCL10) in surviving animals.^20^ Neuroinflammatory activation of glial cells following encephalitic infection could therefore act in concert with neuronal oxidative stress and mitochondrial dysfunction to facilitate protein misfolding and loss of dopaminergic neurons.

Synucleinopathy and gliosis has been reported following infection with WNV and H5N1 influenza A.^29,45,72^ It has been suggested that α-synuclein can restricts replication of RNA viruses in the brain following encephalitic infection.^27^ However, whether aggregation of α-synuclein is a pathological consequence of encephalitic infection or a protective mechanism to restrict replication of viral RNA is still unclear. In the current study, we observed the formation of large endogenous proteinase K-resistant α-synuclein aggregates in the hippocampus and entorhinal cortex, with smaller P129-immunopositive inclusions in the SNpc in mice surviving intranasal infection with WEEV (Fig. 6). We contribute the lack of P129 + in the SNpc likely due to the low expressional levels of SNCA in wild-type mice. Interestingly, we did not detect α-synuclein protein aggregates at 4 DPI, suggesting that development of these pathological protein aggregates was secondary to other sequela of viral infection, such as oxidative stress and inflammatory activation of glial cells. P129 + inclusions appeared to overlap the same neuroanatomical regions that were positive for DsRed following intranasal infection (Fig. 2). Recent studies have shown a very similar distribution of α-synuclein protein aggregates following stereotactic administration of preformed α-synuclein fibrils (PFFs).^73^ Following stereotactic administration of PFF into the olfactory bulb, pK-resistant protein plaques were detected in the hippocampus, cortical regions and substantia nigra at 6 months post injection.^73,74^ Here, we show that the majority of P129 + inclusions did not appear to co-localize with neurons or astrocytes but seemed to be primarily colocalized with IBA1 + microglia. The P129 + cells appeared to be dying neurons which likely no longer express TH and NeuN, explaining the lack of NeuN in P129 + cells. In addition, phagocytosis of dying neuronal populations likely explains the localization of P129 + in microglia, which is consistent with other findings.^75^ Additional studies examining the timecourse of glial activation and protein aggregation will be required to more precisely determine how activation of astrocytes and microglia is linked to aggregation of α-synuclein during infection with WEEV. Reports using stereotactic administration of PFFs into the olfactory bulb did not determine if α-synuclein aggregates in the midbrain spread through direct transneuronal propagation of the originally injected PFFs or from a seeding event that caused the formation of new endogenous α-synuclein aggregates.^73^ Considering that McFly solely expresses firefly luciferase and that no exogenous α-synuclein was introduced during inoculation, our data suggest that seeding of α-synuclein aggregation following infection with WEEV facilitates the pathological conversion of native α-synuclein into the formation of proteinase K-resistant aggregates in select brain regions. Whether these inclusions spread through a transneuronal mechanism or from glia transmission is still unknown. The presence of α-synuclein in IBA1 + cells is likely facilitating the clearance of protein inclusions from the CNS, as previously noted, but additional longitudinal studies are needed to confirm this finding.^74,75^

Common features of neurodegenerative diseases include neuronal loss, neuroinflammation, gliosis, ER-stress, mitochondrial perturbations, autophagy dysfunction, and activation of cell death pathways. We observed significant differences in gene profiles from whole-brain homogenates between surviving host at 3 and 28 DPI. The most significant differences between gene families were involved in lipid metabolism, calcium signaling, inflammation and proteins involved in neurodegeneration. Upregulation of innate immune and cell death modulators was observed at 3 DPI, consistent with a gene profile following acute encephalitic infection. Conversely, we observed a drastic gene profile shift at 28 DPI that produced a pattern of gene expression consistent with a neurodegenerative phenotype: App (amyloid precursor protein), Htt (Huntington), and Park7 (DJ1), which is linked to preservation of mitochondrial function in Parkinson’s disease.^76^ Recent evidence has suggested that Phospholipase D1/D2 (*Pld1, Pld2*) significantly modify autophagy during nutrient deprivation and are direct downstream targets of amyloid precursor protein (APP), possibly explaining the significant upregulation of these genes (Supplementary Fig. 4).^33^ Activation of surrounding glia is likely a key contributor as well, similar to that described in other encephalitic infections causing severe glial activation and release of glial-derived neurotoxic mediators.^70^ Aldh1a1, an astrocytic differentiation marker, was significantly increased in the 28 DPI group. Moreover, NF-κB-dependent release of astrocyte-specific complement proteins has been shown to produce neuronal ER-stress and intracellular neuronal calcium perturbations.^69,70^ Intracellular calcium perturbations and glial activation therefore likely influence the specificity of neuronal injury in the SNpc following infection with WEEV.^77^

In accordance with previous clinical findings, these data demonstrate that encephalitic infection with WEEV in outbred CD-1 mice targets multiple brain regions affected in neurodegenerative diseases such as PD.^13^ To replicate human cases of encephalitic infection with WEEV and to interrogate the long-term neurological consequences ensuing from such infections, immunotherapy treatment was used to establish a cohort of mice that consistently survive CNS infection with WEEV and completely clear virus. The onset, progression and clearance of the infection was demonstrated by in vivo bioluminescence and fluorescent imaging of virally expressed firefly luciferase and dsRed. Examination of surviving mice revealed significant gait abnormalities, loss of dopaminergic neurons in the SNpc and VTA, loss of TH-positive terminals in the striatum, long-lasting glia activation, the formation of proteinase K-resistant α-synuclein inclusions and a gene expression profile consistent with neurodegeneration. The selective and rapid loss of TH + dopamine neurons and development of proteinase K-resistant α-synuclein aggregates from a single inoculation with WEEV, coupled with high specificity for the SNpc, may help to explain why encephalitic infection with mosquito-borne alphavirus can cause parkinsonism in humans. Further work is required to determine how dopaminergic neurons in the SNpc appear to be selectively vulnerable to infection with WEEV. Consistent with clinical cases of encephalitic infection with this virus, the experimental results presented here indicate that the pattern of neuropathological injury and progression of neurodegeneration following infection with WEEV resembles certain features of parkinsonism. It is particularly striking that infection with WEEV caused the development of α-synuclein aggregates within 8 weeks of infection in wild-type mice. This finding, combined with rapid progression, glial activation, and selective loss of dopaminergic neurons within the SNpc suggest that recombinant WEEV, when used in conjunction with immunotherapy, could be a robust animal model for studying neurodegenerative mechanisms relevant to PD.

## Methods

### Viruses

A full-length infectious clone of the WEEV McMillan strain was derived from an isolate obtained from the Arbovirus Reference Collection at the Center for Disease Control and Prevention in Fort Collins, CO, USA, as previously described.^78^ Detailed descriptions of the molecular cloning methods used to construct recombinant WEEV reporter viruses have been previously reported.^9,20^ In brief, duplication of the subgenomic promoter (SGP) sequence (nucleotides 7341–7500 of viral genome) of WEEV McMillan strain was used to express of firefly luciferase. The resulting virus we termed “McFly”. A separate recombinant WEEV was generated that expressed the fluorescent protein, DsRed, and referred to here as “McRed” virus. The McFly and the McRed plasmids were purified by QIAprep Spin MiniPrep Kit (Qiagen, Valencia, CA, USA) and genomic RNA was transcribed in vitro using a T7 RNA polymerase (MAXIscript™ kit, Life Technologies, Grand Island, NY, USA). BHK-21 cells (2 × 10^7^ in 400 µL) were transfected with 20 µL of genomic RNA using an ECM 630 electroporator (BTX Harvard Apparatus, Holliston, MA, USA). Two pulses of 450 V, 1200 Ω, and 150 µF were administered. The rescued virus was passaged once in BHK-21 cells, collected at 48 h post infection (HPI) and stored at −80 °C. Stock viruses were quantified using plaque titration in Vero cells before experimental use. Virus titrations were performed in duplicate and plaque assays were performed as described by Liu and colleagues.^79^

### Mouse infections and in vivo imaging

Mouse infections and imaging analysis were adapted from previous studies.^9^ In brief, all animal protocols used in the current study were approved by the Animal Care and Use Committee at Colorado State University (Permit #11-2605A), and mice were handled in compliance with the PHS Policy and Guide for the Care and Use of Laboratory Animals. All infections were conducted in certified biosafety level 3 (BSL-3) facilities at the Infectious Disease Research Center at Colorado State University in compliance with NIH guidelines under an approved IACUC protocol and were supervised by institutional veterinary staff. Male and female (6-week-old) CD-1 mice (Charles River Labs, Wilmington, MA, USA) were used in this study. Mice were anesthetized with isoflurane (Minrad Inc, Bethlehem, PA, USA) through an XGI-8 anesthesia system (Caliper Life Sciences, Waltham, MA, USA) connected to the IVIS 200 (Caliper Life Sciences, Waltham, MA, USA) imaging camera imaging system. Intranasal infection was conducted at a dose of 1 × 10^4^ PFU in a volume of 20 µL, delivered drop-wise onto the nostrils of lightly anesthetized mice. Mice then received subcutaneous administrations of luciferin substrate (150 mg/kg) to the dorsal cervical spine at 10–15 min before imaging. Exposure time was consistent for all animals. Living Image 3.0 software (Caliper Life Sciences, Waltham, MA, USA) was used to analyze and process images taken using the IVIS 200 camera. Uninfected mice were used as an imaging control to adjust for background signal, and BLM threshold was established using negative imaging controls at 5 × 10^3^ p/s/cm^2^/sr. A standard region of interest was created to measure total light emission for each mouse. Sagittal head images were created by injecting mice subcutaneously with two doses of (150 mg/kg) 10 min apart prior to euthanasia. Animals were then decapitated under isofluorance anesthesia and whole-heads bisected along the medial-sagittal plane, rinsed with PBS and promptly imaged.

### CLARITY method of tissue-transmutation and immunostaining

Briefly, brain tissue was processed for CLARITY imaging by incubating formalin-fixed tissue in a solution of acrylamide monomers and a temperature-activated cross-linker for 3 days.^80^ Tissue was then heated to 40 °C, resulting in the formation of a tissue-hydrogel hybrid. Clearing occurs through the gradual removal of endogenous lipid(s) over several days, using multiple changes of sodium dodecasulfate (SDS). Upon completion of the clearing process, the brains were washed three times in 1× TBS, each wash lasting 2 days. The resultant ‘clarified’ brain tissue was then amenable to 3D imaging using confocal microscopy. Lipid-cleared tissue-hydrogel was sectioned (coronal or sagittal) and treated with primary antibody (anti-TH 1:200) and incubated for 2 days at 4 °C. Primary antibody was washed with Tris-buffered saline for 2 days at room temperature, changing the wash buffer three times over the 2 days. The secondary antibody (anti-rabbit AlexaFluor 488, 1∶2000 dilution) was incubated for 2 days at room temperature. Sections were washed with Tris-buffered saline for 2 days at room temperature, changing the wash buffer three times over the 2 days. Slides were counterstained with Hoechst (1:2000) to identify all nuclei. Sections were then washed for 30 min and then mounted with a coverslip using 80% glycerol in TBS.

### 3D-reconstruction of the mouse brain from caudal olfactory to rostral cerebellum

CD-1 mice were infected by intranasal or footpad route with 10^4^ PFU WEEV. At first sign of neurological disease, mice were euthanized and processed using the CLARITY method, as described above. Tissue sections were counterstained with DAPI (1:2000), washed for 30 min, mounted using 80% glycerol in tris-buffered saline (TBS), and imaged. The full section montage images resulting from CLARITY imaging were deconvoluted and processed to obtain 3D reconstructions using Imaris software (Bitplane AG, Zurich, Switzerland). Due to the technical constraints of imaging such large volumes of tissue, our imaging was limited to the region from the caudal olfactory to the rostral cerebellum, the regions most relevant to parkinsonism.^18^

### Tissue preparation and immunofluorescent staining

Tissue processing and immunofluorescent staining for dopamine neurons, glial cells and phosphorylated α-synuclein (p129) was performed as reported previously.^29,81,82^ In brief, animals were terminally anesthetized with isoflurane and transcardially perfused. After perfusion fixation, the brains were carefully removed from the skull and immersed in the same fixative at 4 °C for 3 h. The brains were then equilibrated in cacodylate-PBS containing 15% sucrose overnight, followed by 30% sucrose. The tissue was then embedded and sectioned at 40 microns on a cryostat microtome producing a mean of 60 sections through the anatomic SN nucleus. Sections were stored at –20 °C, free-floating in cryoprotectant (30% w/v sucrose, 30% v/v ethylene glycol; 0.5 M phosphate buffer; pH 7.2) until staining. Free-floating serial sections were washed in PBS and mounted on glass slides for staining followed by antigen retrieval with 0.01 M sodium citrate buffer (pH 8.45) for 20 min before blocking. All primary antibodies were diluted to their optimized dilutions in 0.1% Triton-X containing phosphate buffered saline (PBS): rabbit tyrosine hydroxylase (TH; Millipore; 1:500), mouse Neuronal Nuclei (NeuN; Millipore; 1:250), chicken microtubule-associated protein 2 (MAP2; Abcam 1:100), rabbit glial fibrillary acid protein (GFAP; Dako, 1:500), rabbit Ionized calcium binding adaptor molecule 1 (Iba1; Wako; 1:250), goat ionized calcium binding adaptor molecule 1 (Iba1; Abcam; 1:50), and mouse anti-alpha-synuclein (P129, Wako, 1:100). Sections were stained for DAPI (Sigma) and mounted on glass coverslips in VectaShield mounting medium and stored at 4 °C until imaging.

### Cell counting of neurons and glia in the midbrain, hippocampus, and cortical brain regions

Methodologies for imaging and counting of dopamine neurons and glial cells in the SN and nerve terminal in the striatum were adapted from those previously reported.^83^ Every sixth tissue section was selected and counted, for a total of eight sections per animal for dopaminergic cells and a total of two sections per animal for glia. The studies described here were all performed blinded by a single investigator. Images were captured using an automated Olympus BX51 fluorescence microscope equipped with a Hamamatsu ORCA-flash 4.0 LT CCD camera and collected using Olympus Cellsens software (v 1.15). Quantitative analysis was performed on dual- or triple-labeled fluorescent images generated by montage imaging of an entire coronal brain section compiled from individual images acquired using an Olympus PlanApochromat ×10 air objective (0.40 N.A.). One hemisphere of the SN was delineated as an active ROI on the basis of TH immunolabeling and with reference to a coronal atlas of the mouse brain (Allen Brain Atlas). All slides were scanned under the same conditions for magnification, exposure time, lamp intensity and camera gain.

For quantitative assessments, TH +, NeuN +, IBA1 +, and GFAP + soma from the selected ROIs were automatically detected using the CellSens dimension platform. Area of soma, mean intensity values and shape factor threshold-based based quantification filters were manually adjusted to acquire accurate TH +, NeuN +, IBA1 +, and GFAP + cell body counts. Once all eight sections of a single brain were counted for neurons, the total number of TH + and NeuN + neurons counted were multiplied by the reciprocal of the volume fraction (18), which is mathematically derived from three variables; area sampling fraction (1), the height sampling fraction (30 μm/40 μm), and the section sampling fraction (1/6). Mathematical derivations for neuronal counts were adapted from previously cited methods.^83^ Two sections of each brain were counted for glia and the total number of IBA1 + and GFAP + glia for each section were divided by the area of the ROI (mm^2^) to normalize across all brains. Representative montage images were generated for each treatment group with use of a ×10 air PlanApochromat objective (0.40 N.A.). Anatomic landmarks were used to select striatal sections for tyrosine hydroxylase intensity staining in an identical process as we previously reported,^84^ staining all treatment groups simultaneously. Montage images of the striatum were created using a ×10 objective and a mask generated to outline the striatum with use of Slidebook software, and mean fluorescence intensity in relative florescence units was obtained. Representative montage images were generated for each treatment group with use of a ×10 objective and were displayed using inverted monochrome.

To determine hippocampal and entorhinal cortex total neuronal densities, brains from McRed-infected and age-matched CD-1 control mice were serially sectioned at 10 μm. Sections were quantified as previously preformed.^85^ In brief, 10 μm sections of brain were stained with Cresyl violet to identify Nissl-positive cells. At least two sections from the rostral hippocampus containing a fully intact CA1 region (Paxinos and Franklin Bregma −1.70 to 2.18) as well as entorhinal cortex (Bregma −3.52 to −4.04) were identified. For hippocampal densities, the CA1 region medial to the faciola cinereum to CA2 was outlined at 4× using the Neurolucida Neuron Tracing Program. Once the CA1 was outlined, the area of CA1 was empirically determined based on the Neurolucida calibration tool. To determine the cell number, all pyramidal neurons in CA1 were counted at 40 ×. The number of cells within CA1 were then divided by area to determine cells/μm^2^. At least two sections/rostral hippocampus were counted in each brain and an average of these densities was determined. A similar method was used to determine the cellular density of the entorhinal cortex.

### Generation of anti-alphavirus E1 immune serum

For preparation of E1 serum, rabbits were vaccinated with recombinant WEEV McMillan strain E1-ectodomain antigen (10 μg antigen/dose), polyI:C (dsRNA analog), and ODN 1826 (unmethylated CpG DNA) (InVivoGen) to a final concentration of 0.1 mg/mL each, in TiterMax Gold adjuvant (Sigma, St. Louis, MO). Each element was added to the aqueous medium before emulsion with TiterMax Gold prior to injection. This priming dose was followed by an identical boost vaccination, every 2 weeks, until a total of four doses were administered. Nine weeks following the last boost dose, rabbits were terminated and serum was collected, heat inactivated at 56 °C for 30 min and stored at −80 °C. Naive serum was collected from control animals. An aliquot of immune serum was tested for titer against E1 antigen-coated titration plates and found to have an antibody titer measurement >26,000 reciprocal value, the cut-off for the assay.

### Mouse gait analysis

Mouse gait analysis was performed as previously published.^81^ Two weeks before initial behavioral testing, all mice were acclimated to the gait analysis equipment and baseline gait measurements were taken. Gait measurements of step size, rate and paw intensity were detected using a custom made real-time video gait analysis system. Video recordings of unrestricted movement down a 1 m glass trackway were conducted measure gait parameters, including stride length, as previously reported.^81^ All gait testing was performed 8 weeks after inoculation in mock-infected control mice and in mice infected with WEEV. Values for each parameter were subtracted from initial values at day 0 to obtain measurements representing the change from baseline and all metric were compared back to control/mock-infected mice.

### Proteinase K digestion of frozen tissue sections

For the detection of α-synuclein aggregation in WEEV challenged mice in intact brain sections, free-floating brain sections from infected and non-infected mice were mounted on charged glass slides and probed for phosphorylated α-synuclein (PhosphoSer129/p129) following digestion with proteinase K. Sections were then washed 3 × 5 min with TBS and allowed to adhere onto charged slides for 15 min. Sections were then subjected to antigen retrieval by incubating in 0.01 M sodium citrate buffer (pH 8.45) for 20 min. Proteinase K (pK) (Roche 03-115-887-001) was added to each section at a concentration of 100 µg/mL for 30 min at 55 °C. Sections were again washed 3 × 5 min with TBS and then blocked with either 1% donkey serum or a combination of 1% donkey serum and 1% goat serum. Primary antibodies diluted in 0.1% Triton-X containing TBS are as follows: Rabbit polyclonal anti-tyrosine hydroxylase (1:500; Millipore Cat# AB152), mouse monoclonal anti-p129 (1:100; Wako Cat# 015-25191), and goat polyclonal anti-IBA1 (1:50; Abcam ab5076). Primary antibody was incubated on the sections for 24 h in 4 °C. Secondary antibodies utilized were diluted in TBS along with 1% donkey serum and are as follows: Donkey anti-rabbit Alexa488 (1:500; ThermoFisher A21206), donkey anti-mouse Alexa555 (1:500; ThermoFisher A31570), and donkey anti-goat Alexa647 (1:500; ThermoFisher A21447). Sections were counterstained with DAPI (1:2000) and mounted on glass coverslips in VectaShield mounting medium and stored at 4 °C until imaging.

### Transcriptional analysis of whole-brain homogenates

Dissected whole brains (*n* = 3/group) was homogenized and solubilized using TRIzol® Reagent (ThermoFisher Scientific) and RNA purified per manufacturer’s instructions. The purified total RNA was converted to cDNA using the RT^2^ first strand kit (SABiosciences) and analyzed using the following SABiosciences (Qiagen) qPCR arrays: (1) PAMM-098Z Mouse mTOR Signaling PCR Array, (2) PAMM-212Z Mouse Cell Death Pathway Finder PCR Array, and (3) PAMM-124Z Mouse Parkinson’s Disease PCR Array. All arrays were read using a BioRad iQ5 real-time PCR detection system. The same amount of cDNA was applied to each array for (1) uninfected control animals, (2) infected animals, or (3) infected and immunotherapy-treated animals. Gene expression data was normalized to the internal control genes, beta-2 microglobulin and glyceraldehyde-3-phosphate dehydrogenase across all arrays for each experimental condition based on the criteria of minimal Ct variance, as reflected by lowest standard deviation, and highest normality of distribution.^22^ Normality of distribution was determined using JMP Version 8 (SAS Institute) statistical software.

### Reporting summary

Further information on research design is available in the Nature Research Reporting Summary linked to this article.

## Supplementary information

Supplementary Information

Supplemental Video 1

Supplemental Video 2

Supplemental Video 3

Reporting summary

## Data Availability

The authors of this paper declare that all the data supporting the findings of this study are available within the paper and available in the supplementary figure files. Any additional, raw values may be obtained from the corresponding author, Prof. Ronald Tjalkens, upon request.

## Supplementary information

**Supplementary information** accompanies the paper on the *npj Parkinson’s Disease* website (10.1038/s41531-019-0090-8).
