# Supplementary material for: Infection with mosquito-borne alphavirus induces selective loss of dopaminergic neurons, neuroinflammation and widespread protein aggregation
Source: NPJ Parkinsons Dis. 2019 Sep 13;5:20. doi: 10.1038/s41531-019-0090-8 (PMC6744428; doi:10.1038/s41531-019-0090-8)
Supplement: Supplementary file 1 — Supplementary Information [file 41531_2019_90_MOESM1_ESM.pdf]

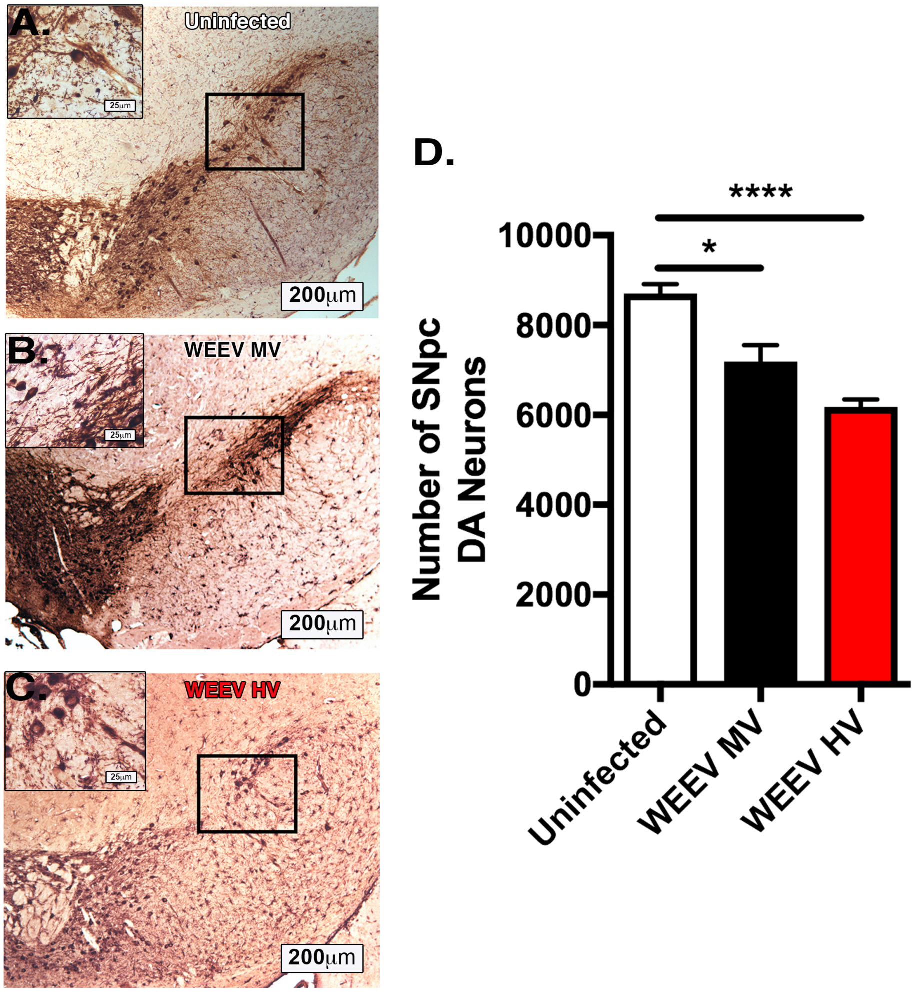

Supplemental Figure 1. High-virulence (McMillan strain) causes more significant dopaminergic neuronal in loss in the SNpc than medium-virulence (Montana-64 strain) within 4 days of infection. (A-C) Six-week old CD-1 mice were administered 1×10^4 PFU medium virulent wEEV/Montana-64 strain (MV) or high virulent WEEV/McMillan (HV) or mock infected via intranasal inoculation and euthanized four days post-infection. (**D)** Brains were cryosectioned and the number of DA neurons in SNpc were examined with 3D design-based stereology 4 DPI. (N=3/group). * $p<.05$ and ****$p<0.0001$.

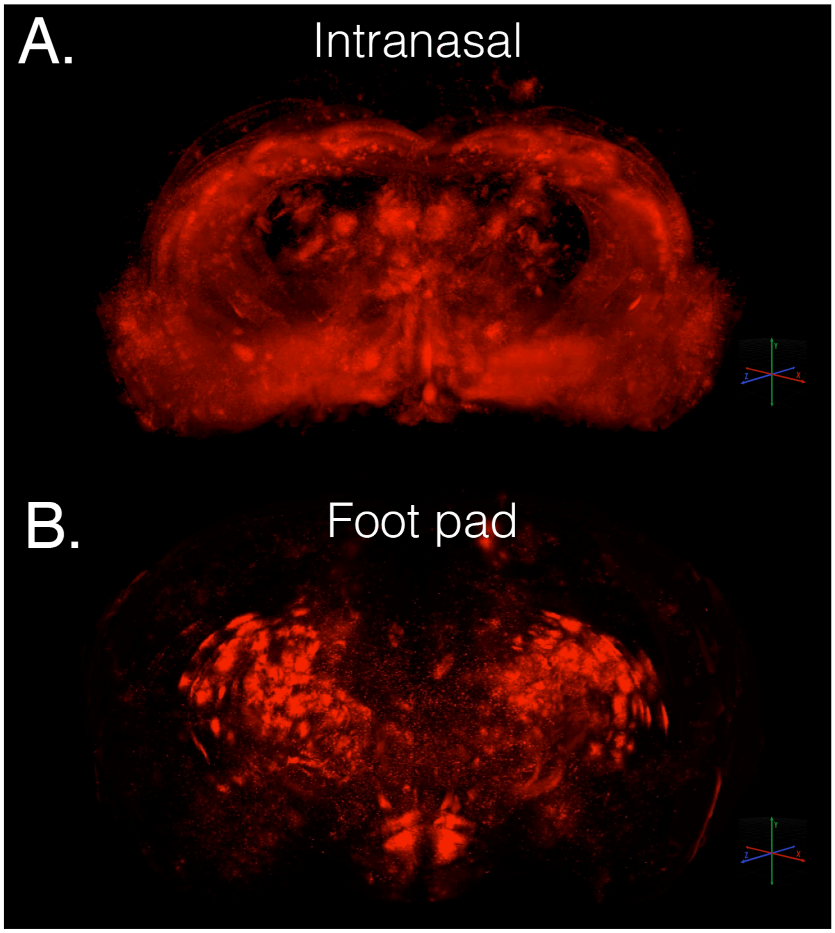

Supplemental Figure 2. Infection with McRed by intranasal and subcutaneous foot pad inoculation result in dramatically different patterns of viral dissemination in the CNS. (See Supplemental Videos 1 and 2) 3D reconstructions of dsRed-expressing WEEV in brain following intranasal (**A**) or foot pad (**B**) routes of inoculation. Six-week old CD-1 mice were administered 1×10^4 PFU of McRed (McMillan-DsRed) via intranasal inoculation and euthanized four days post-infection. Brains were perfused, embedded in hydrogel and sectioned at 200 μM. Sections were then cleared, removing all lipids, and montages at 10x magnification. 3D-reconstructions were built using Imaris software.

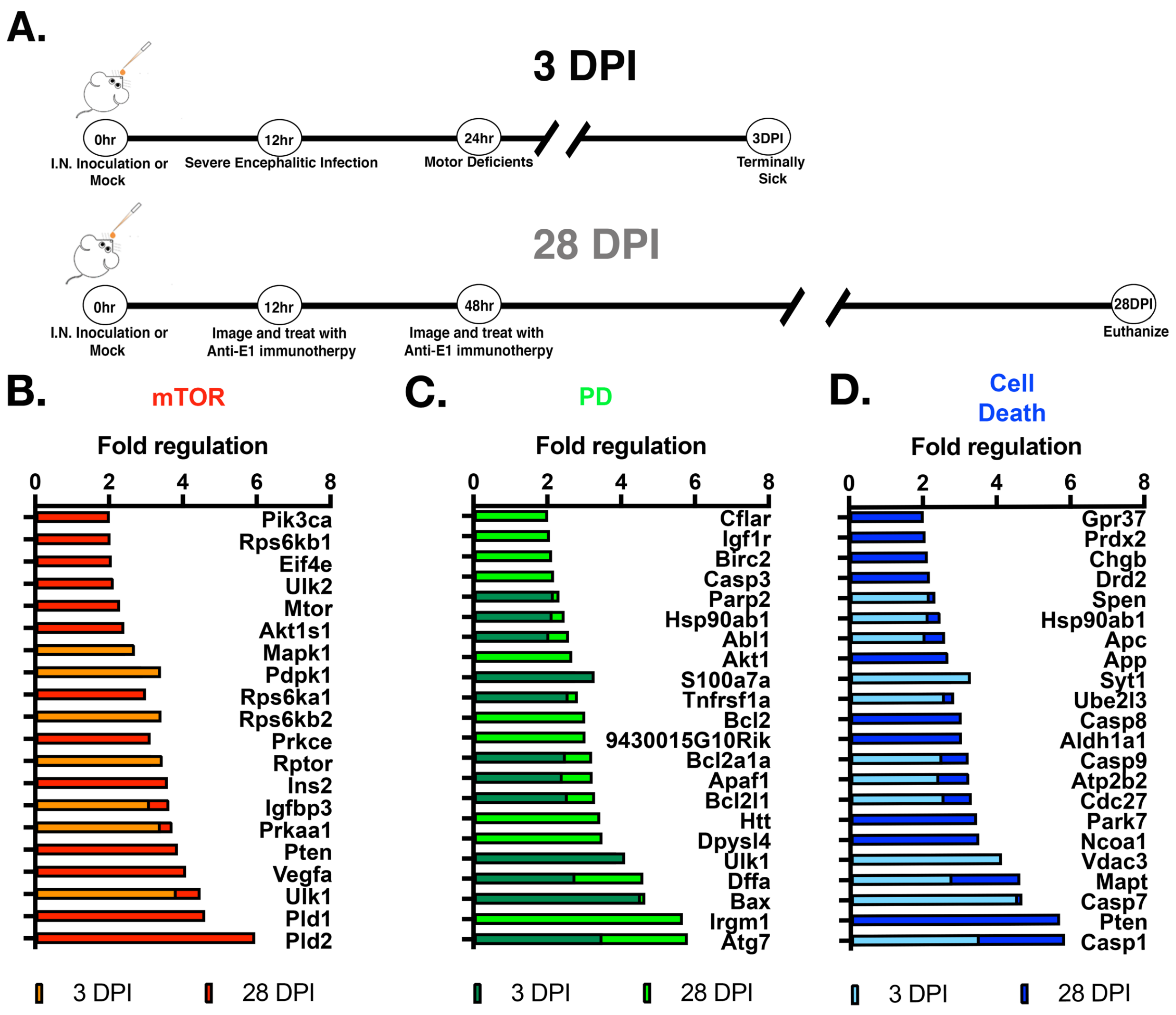

Supplemental Figure 3. Infection with WEEV causes a gene expression profile consistent with neurodegeneration. (A) Treatment schematic. RNA was extracted from whole brain homogenate and assayed using three separate pathway-specific RT² Profiler PCR Array plates: **(B)** mTOR pathway, **(C)** PD-specific genes, and **(D)** Cell Death Pathway Finder. WEEV-infected mice were normalized to mock-infected/IgG-treated control mice. (N=4/group).

Supplemental Video 1. Viral dissemination throughout the CNS following infection with McRed by intranasal inoculation. 3D reconstruction of dsRed-expressing WEEV in brain following intranasal inoculation was based on montage images of CLARITY brain sections (200 um) collected rostral – caudal throughout the entire mouse brain at 4 days post-infection. Reconstruction and rendering was performed using Bitplane software (IMARIS).

Supplemental Video 2. Viral dissemination throughout the CNS following infection with McRed by foot pad inoculation. 3D reconstruction of dsRed-expressing WEEV in brain following foot pad inoculation was based on montage images of CLARITY brain sections (200 um) collected rostral – caudal throughout the entire mouse brain at 4 days post-infection. Reconstruction and rendering was performed using Bitplane software (IMARIS).

Supplemental Video 3. Locomotor deficits in CD-1 mice following infection with WEEV (McRed) at 4 days post-infection.
